# Pharmacological effects of statins in adult patients with type 2 diabetes mellitus: A protocol for systematic review and meta-analysis

**DOI:** 10.1097/MD.0000000000032313

**Published:** 2022-12-23

**Authors:** Kabelo Mokgalaboni, Phiwayinkosi V. Dludla, Bongani B. Nkambule

**Affiliations:** a Department of Life and Consumer Sciences, College of Agriculture and Environmental Sciences, University of South Africa, FL, South Africa; b School of Laboratory Medicine and Medical Sciences (SLMMS), College of Health Sciences, University of KwaZulu-Natal, Durban, South Africa; c Biomedical Research and Innovation Platform, South African Medical Research Council, Tygerberg, South Africa.

**Keywords:** endothelial dysfunction, endothelium, inflammation, statins, type 2 diabetes mellitus

## Abstract

Due to contradicting findings on impact of statins on endothelial function in type 2 diabetes mellitus especially across the randomized controlled trials (RCTs). With this systematic review, we aim to evaluate whether the use of statins improves endothelial function in adults with type 2 diabetes. We will further highlight if these biomarkers are ideal therapeutic targets for risk for atherosclerosis and cardiovascular disease.

**Methods:**

This protocol was carried out according to the preferred reporting items for systematic review and meta-analysis protocols-2015 guideline. The online databases, such as MEDLINE, Scopus, and Web of Sciences, will be targeted using the medical subject heading terms (MeSH) and text words. The review will include clinical studies on the effect of statins on markers of endothelial function in type 2 diabetes. The Cochrane risk of bias guideline will be used to assess the quality and risk of bias. We are planning to use the grading of recommendation assessment, development, and evaluation approach to evaluate the strength and quality of evidence.

**Results:**

This study will not involve human samples and patient data; hence ethics approval will not be required. The findings will be presented in journal clubs and conferences and published in peer-reviewed journals.

## 1. Introduction

Type 2 diabetes mellitus (T2D) is a chronic metabolic disorder associated with impaired insulin metabolism and hyperglycemia. According to a 2021 International Diabetes Federation (IDF) report, about 537 million people had diabetes which is anticipated to increase to 643 and 783 million by 2030 and 2045, respectively.^[1]^ Due to this increasing rate of diabetes globally, the risk of cardiovascular disease (CVD) and death due to diabetes-related complications is also alarming. It is well documented that endothelial dysfunction appears as the first vascular disorder in T2D and is also observed in pre-diabetes.^[2,3]^

Notably, in a normal state, there is a balance between producing and releasing vasoconstrictors and vasodilators from the endothelial lining. This helps maintain a healthy endothelial system and is attributable to its antioxidant, anti-inflammatory, and anti-thrombotic effects.^[4–6]^ However, this homeostatic mechanism is impaired in T2D, resulting in endothelial dysfunction, which is a central finding in atherosclerosis, and it is demonstrated by imbalanced vasodilation, vasoconstriction, oxidative stress, and pro-inflammatory factors, and reduced level of nitric oxide (NO).^[7–9]^ A previous review by Janus and colleagues reported that insulin resistance in T2D promotes endothelial dysfunction, and the latter promotes the development of atherosclerosis and other CVDs.^[10]^ T2D is associated with reduced flow-mediated dilatation (FMD),^[11]^ a noninvasive approach for the assessment of endothelial function.^[12,13]^ However, its impairment suggests an imbalanced production of vasodilators, hence endothelial dysfunction.

Several adhesion molecules and markers are expressed when the endothelial lining is impaired; these markers include intercellular adhesion molecule-1, 2, vascular cell adhesion molecule-1 (VCAM-1), endothelial microparticles, E and P-selectin.^[14–17]^ Most importantly, the expression of these markers on endothelial cells would suggest an endothelial cell injury; therefore, they can be used as ideal biomarkers for endothelial function and the evaluation of risk for CVD amongst T2D. However, leukocytes also express P-selectin, suggesting that its expression would not be an ideal marker for endothelial function.

A wide population of T2D relies on metformin and statins against secondary conditions such as atherosclerosis and CVD. Previous evidence shows the beneficial impact of metformin on endothelial function.^[18–21]^ Apart from its role as a lipid-lowering drug, statins have contributed to the maintenance of endothelium health.^[22,23]^ However, its combination therapy with metformin in T2D patients has proven to have adverse effects.^[24]^ While the results from in vivo and in vitro studies show the beneficial impact of statin on endothelial function,^[25–27]^ the same results cannot be reproduced in clinical trials. Of concern are the controversial findings from previous randomized control trials (RCT) that explored the effect of statins on endothelial activation and function in T2D patients. Few studies reported null findings when cerivastatin and atorvastatin were used,^[28–31]^ while others reported an improved endothelial function in T2D patients on atorvastatin treatment.^[32–34]^ In preclinical studies, these beneficial effects are due to the inhibition of the isoprenoid synthesis in a mevalonic pathway associated with an upregulated endothelial NO synthase expression, increased NO production, reduced oxidative stress, plaque stabilization, anti-inflammatory, and anti-thrombotic activity.^[27,35]^

Noteworthy, a recent meta-analysis by Zinelu and Mangoni showed that statin treatment significantly lowered serum concentrations of adhesion molecules in patients with metabolic syndromes,^[36]^ there are concerns related to heterogeneity and the risk of publication bias. In contrast to our proposed review, the results combined in this meta-analysis were derived from studies conducted on patients with various metabolic disorders.

Therefore, it is crucial to investigate the effects of statins on endothelial function in patients with T2D so that better intervention against CVD can be implemented early amongst T2D at high risk. This will inform us whether the downregulation of endothelial activation biomarkers can be targeted when developing alternative treatments. In addition to exploring whether statins in T2D can improve endothelial function based on FMD assessment. In the proposed study, we also aimed to synthesize data from RCTs evaluating the effects of statins in T2D patients with a focus on the selected markers of endothelial function.

## 2. Methods and registration

This protocol has been prepared and reported using the Preferred Reporting Items for Systematic Review and Meta-Analysis Protocols 2015 guideline^[37]^ (Supplementary File 1, Supplemental Digital Content, http://links.lww.com/MD/I127). This protocol requires no ethics approval from the ethics committee as it will involve published data. This protocol is registered with the International Prospective Register of Systematic Reviews (PROSPERO), registration number: CRD42021178350. https://www.crd.york.ac.uk/prospero/display_record.php?ID=CRD42021178350.

### 2.1. Ethics approval

This study will not require ethics approval from the ethics committee; this proposed study will synthesize the evidence from already published studies.

### 2.2. Objective

To evaluate whether statin therapy can downregulate the expression of markers and improve endothelial function in adult patients with T2D.

### 2.3. The eligibility criteria of this review will follow the PICO standards

#### 2.3.1. Population.

Adult patients aged 18 years and above living with T2D.

#### 2.3.2. Intervention.

Statins (atorvastatin, fluvastatin, pitavastatin, pravastatin, rosuvastatin, simvastatin, cerivastatin, and lovastatin).

#### 2.3.3. Comparator.

1. T2D adult (18 years and above) patients on a placebo.

2. Healthy participants that served as control.

#### 2.3.4. Outcome.

Endothelial function.

#### 2.3.5. Effect measures.

Vascular cell adhesion molecule-1, intercellular adhesion molecule-1 and FMD.

#### 2.3.6. Study design.

Randomized controlled trials (RCTs).

#### 2.3.7. Information source and search strategy.

The search strategies will be developed using MesH terms on MEDLINE, Cochrane library, Web of Science, and Scopus text words. A comprehensive search will be conducted for human studies published from inception until 2022 with consultation with the librarian. Additionally, manual screening of references of selected studies will be performed to identify relevant studies missed through a database search. The major MeSH terms and synonyms will comprise of “statin,” OR “hydroxymethylglutaryl coenzyme A reductase inhibitor,” OR “Hydroxymethylglutaryl coenzyme-A reductase inhibitor,” OR “atorvastatin,” OR” fluvastatin,” OR “simvastatin,” OR “pitavastatin,” “pravastatin,” OR “lovastatin,” OR “cerivastatin,” OR “rosuvastatin,” AND “endothelial,” OR “endothelium,” AND “type 2 diabetes mellitus,” OR “type 2 diabetes,” OR “hyperglycemia.” There will be no language restriction, and studies published in other languages will be translated using google Translate. The exact strategy attached is presented in Supplementary File 2, Supplemental Digital Content, http://links.lww.com/MD/I128.

### 2.4. Study selection procedure

Two (KM and PVD) investigators will independently perform study selection to minimize the risk of selection bias. Each investigator will initially screen the titles, abstracts, and keywords guided by our PICO and eligibility criteria. This will be followed by retrieving full-text studies and further screening of retrieved studies. The studies reporting on data from the same trial and those retracted from journals will all be excluded. Investigators will request a full-text study from the corresponding authors if the studies are not available on the database. Should the corresponding author not provide an article, this will be excluded from the analysis. Most importantly, Cohen’s Kappa inter-rater reliability will be used to evaluate the level of agreement between the 2 investigators.^[38]^ In case of a high level of disagreement, as noted by Cohens Kappa results, the third independent investigator (BBN) will evaluate the study in dispute and make a conclusion.

### 2.5. Eligibility criteria will be based on the following considerations

1. RCTs will be included if conducted in adult (18 years and above) patients with T2D on statin treatment.
2. RCTs with control groups as T2D on placebo or healthy patients will be considered.
3. Studies conducted on children will be excluded.
4. Studies on animal models or experimental studies on T2D will not be considered.
5. Studies in T2D patients on statins with other treatments will be excluded.
6. Results from unpublished studies or retracted studies, reviews, and conference abstracts will not be excluded.

### 2.6. Data management

#### 2.6.1. Data collection and items.

Two independent investigators (KM and BBN) will extract data from the relevant studies to avoid discrepancies and inconsistencies in the data extraction process. These investigators will independently collect data from all studies using a designed PICO guideline and a standard data extraction sheet using an Excel spreadsheet. The data items will include author names and year of publication, the study’s location, study design, population, sample size, gender and age, type of treatment and dosage used, markers of endothelial function, and main findings. Mendeley Reference Manager Desktop, Elsevier, Amsterdam, the Netherlands (Version 1.19.4), will be used to save studies from the database and identify duplicates. In case of discrepancies and inconsistencies, the third investigator, PVD, will assess the study or data in question and arbitrate.

#### 2.6.2. Data simplification.

As a data simplification measure, all studies indicating that diabetic patients were on statin treatment will be grouped as the treatment group, regardless of the form of statin used, and those with diabetes on a placebo be classified as the control group. Additionally, some studies may report different doses of statins used. To minimize potential bias from comparisons of different statin doses with a single control group, treatment doses will all be combined into a single statin treatment group. Furthermore, the final analysis will combine studies that report on the same outcomes.

#### 2.6.3. Risk of bias in individual studies.

To evaluate the potential risk of bias across all included studies, the Cochrane Risk of Bias Assessment Tool will be used.^[39]^ This tool assesses 6 key domains: random sequence generation, allocation concealment, blinding of participants and personnel, blinding of outcome assessment, incomplete outcome data, and selective reporting. Two independent investigators will judge each domain as having a low risk of bias, high risk of bias, or unclear risk of bias. Consistently, the overall quality of each study will be classified as excellent if judged as low risk in about 4 to 6 domains, fair if it has 3 domains judged as low risk, and poor if it has 2 or less domains judged as low risk.

### 2.7. Statistical analysis

We will perform meta-analyses only if adequate RCTs (more than 2) have reported or provided estimates of the outcomes of interest. Otherwise, a qualitative approach of available evidence will be undertaken. All data analysis will be performed using Review Manager software version 5.4.1 (The Nordic Cochrane Centre, The Cochrane Collaboration, 2014). Cochran’s Q statistic^[40]^ and the *I*^*2*^ statistics^[42]^ will be used to analyze statistical heterogeneity between studies.^[41]^ An *I*^*2*^ ˃50%, *I*^*2 *^< 10% will be regarded as moderate and low heterogeneity, respectively. A random and fixed-effects model will be used in instances of significant heterogeneity or no evidence of heterogeneity, respectively.^[26]^ Continuous data will be reported as mean differences or standardized mean differences and a 95% confidence interval (CI) based on the reported units of measurement. A *P* values ≤ 0.05 will be considered statistically significant. A funnel plot will be used to evaluate the risk of publication bias across the studies.^[43,44]^ To evaluate the source of heterogeneity subgroup analysis by comparing study effect estimates following subgroups according to age, gender, population, the region where the study was conducted, the type of statin and dosage, period of intervention, and the overall quality of studies. Kappa values of 0 will be considered as no agreement, 1 as a perfect agreement, and 0.21 to 0.40 will be considered fair agreement.

### 2.8. Sensitivity analysis and publication bias

We will conduct a sensitivity analysis to evaluate the robustness of the findings by excluding studies of high risk and low quality and based on study weight to evaluate the potential sources of heterogeneity (*I*^*2*^* *> 50%). Publication bias across the studies will be assessed visually and graphically through the use of funnel plots; however, about ten studies will be required to evaluate this.

### 2.9. Cumulative evidence

A qualitative approach will be performed if the studies are not sufficiently homogeneous. Evidence from the included studies will be summarized, tabulated, and personal viewpoints be made. Two independent investigators (KM and PVD) will evaluate the consistency of evidence across studies using the grading of recommendations assessment development and evaluation guideline.^[45]^ The guideline will be used to downgrade the studies in accordance with limitations such as inconsistency, indirectness, misinterpretation of results, and publication or reporting bias. The scores will be upgraded for studies with large effect sizes and reported methods for adjusting confounders.^[46]^ The scores for each outcome will be classified as high, moderate, or low, followed by a rating of the overall quality. The summary of findings table (SOF) will be used to present the overall results, and this will be generated using RevMan version 5.4.1.

## 3. Discussion

T2D is associated with an impaired FMD and, thus, endothelial dysfunction, which initiates the development of atherosclerosis and other CVDs. Endothelial activation is characterized by upregulation in the soluble molecules markers. Thus, the downregulation of these biomarkers using any therapy or treatment can help ameliorate atherosclerosis and CVD among T2D patients. The proposed systematic review and meta-analysis will highlight the effects of statins on endothelial function in patients with T2D. In addition, it will explore whether these biomarkers may be used as therapeutic targets in patients with T2D who may be at risk of CVD-related complications.

## Author contributions

**Conceptualization:** KM, PVD, and BBN.

**Formal analysis:** KM.

**Funding acquisition:** PVD and BBN.

**Investigation:** KM.

**Methodology:** KM, PVD, and BBN.

**Resources:** PVD and BBN.

**Software:** KM.

**Supervision:** PVD and BBN.

**Validation:** KM, PVD and BBN.

**Visualization:** KM, PVD and BBN.

**Writing – original draft:** KM.

**Writing – review & editing:** KM, PVD, and BBN.

## Supplementary Material
