# Supplementary material for: Pharmacological effects of statins in adult patients with type 2 diabetes mellitus: A protocol for systematic review and meta-analysis
Source: Medicine (Baltimore). 2022 Dec 23;101(51):e32313. doi: 10.1097/MD.0000000000032313 (PMC9794268; doi:10.1097/MD.0000000000032313)
Supplement: Supplementary file 2 [file medi-101-e32313-s002.pdf]

**Table 1:** Search strategy adapted on MEDLINE, last search 15/10/2022

| Item | MeSH terms | Search | Hits |
|---|---|---|---|
| 1 | Hydroxymethylglutaryl coenzyme a reductase inhibitor | hydroxymethylglutaryl coenzyme A reductase inhibitor [MeSH Terms] | 33154 |
| 2 | HMG CoA reductase inhibitor | HMG CoA reductase inhibitor [MeSH Terms] | 33154 |
| 3 | Statin | Statins [MeSH Terms] | 33154 |
| 4 | Atorvastatin | Atorvastatin [MeSH Terms] | 7158 |
| 5 | Fluvastatin | Fluvastatin [MeSH Terms] | 1444 |
| 6 | Simvastatin | Simvastatin [MeSH Terms] | 8246 |
| 7 | Pravastatin | Pravastatin [MeSH Terms] | 3518 |
| 8 | Rosuvastatin | Rosuvastatin [MeSH Terms] | 2807 |
| 9 | Pitavastatin | Pitavastatin [MeSH Terms] | N/A |
| 10 | Lovastatin | Lovastatin [MeSH Terms] | 11733 |
| 11 | Cerivastatin | (cerivastatin [MeSH Terms]) | N/A |
| 12 | Endothelial | Endothelial [MeSH Terms] | 121499 |
| 13 | Endothelium | Endothelium [MeSH Terms] | 121499 |
| 14 | Type 2 diabetes mellitus | type 2 diabetes mellitus [MeSH Terms] | 158467 |
| 15 | Type 2 diabetes | type 2 diabetes [MeSH Terms] | 158467 |
| 16 | hyperglycaemia | Hyperglycaemia [MeSH Terms] | 39607 |
| 17 | 1,2,3,4,5,6,7,8,9,10 and 11 | ((((((((((hydroxymethylglutaryl coenzyme A reductase inhibitor [MeSH Terms]) OR (HMG CoA reductase inhibitor [MeSH Terms])) OR (Statins [MeSH Terms])) OR (atorvastatin [MeSH Terms])) OR (Fluvastatin [MeSH Terms])) OR (simvastatin [MeSH Terms])) OR (pravastatin [MeSH Terms])) OR (rosuvastatin [MeSH Terms])) OR (Pitavastatin [MeSH Terms])) OR (lovastatin [MeSH Terms])) OR ((cerivastatin [MeSH Terms])) | 45192 |
| 18 | 12, 13 | (endothelial [MeSH Terms]) OR (Endothelium [MeSH Terms]) | 121499 |

| | | | |
|---|---|---|---|
| 19 | 14,15,16 | ((type 2 diabetes mellitus [MeSH Terms]) OR (type 2 diabetes [MeSH Terms])) OR (hyperglycaemia [MeSH Terms]) | 189264 |
| 20 | 17,18,19 | ((((((((((((hydroxymethylglutaryl coenzyme A reductase inhibitor[MeSH Terms]) OR (HMG CoA reductase inhibitor[MeSH Terms])) OR (Statins[MeSH Terms])) OR (atorvastatin[MeSH Terms])) OR (Fluvastatin[MeSH Terms])) OR (simvastatin[MeSH Terms])) OR (pravastatin[MeSH Terms])) OR (rosuvastatin[MeSH Terms])) OR (Pitavastatin[MeSH Terms])) OR (lovastatin[MeSH Terms])) OR ((cerivastatin[MeSH Terms]))) AND ((endothelial[MeSH Terms]) OR (Endothelium[MeSH Terms]))) AND (((type 2 diabetes mellitus[MeSH Terms]) OR (type 2 diabetes[MeSH Terms])) OR (hyperglycaemia[MeSH Terms])) | 75 |

<u>COCHRANE Library: Last search 11/10/2022</u>

Main keywords and Boolean operators used

“Statins” AND “Endothelial function” AND “type 2 diabetes mellitus”

60 records identified
